# Thiol-functionalized ZrP reinforced poly(MMA-co-MI) nanocomposites: High optical transparency and superior thermal stability for optical coatings

**DOI:** 10.1371/journal.pone.0345040

**Published:** 2026-05-19

**Authors:** Shahrzad Jahangiri, Samira Sarikhani

**Affiliations:** 1 Department of Chemistry, College of Science, Malek-ashtar University of Technology, Shahin-shahr, Islamic Republic of Iran; 2 Department of Chemistry, College of Science, Malek-ashtar University of Technology, Shahin-shahr, Islamic Republic of Iran; University of Sharjah, UNITED ARAB EMIRATES

## Abstract

This study presents novel transparent and thermally stable nanocomposites based on poly(methyl methacrylate-co-N-2-methyl-4-nitrophenylmaleimide) (P(MMA-co-MI)) reinforced with thiol-functionalized zirconium phosphate (ZrP-SH) nanofillers. The key innovation is the thiol modification of ZrP, which enhances interfacial interactions and ensures uniform dispersion at low loadings (≤1 wt%). The nanocomposites were prepared through solution blending and characterized using FTIR, XRD, SEM, TEM, TGA, DSC, and UV-Vis spectroscopy. Compared to the neat copolymer, the nanocomposites demonstrated an approximately 30 °C increase in glass transition temperature (from 164.3 °C to 168.4 °C) and an increase in onset degradation temperature from 265 °C to about 297 °C while maintaining high optical transparency in the visible range at low filler contents. These silicon-free materials effectively address the trade-off between thermal stability and optical clarity, offering significant potential for advanced optical coatings, sensors, protective layers, and optoelectronic packaging applications.

## 1. Introduction

Polymeric materials are widely used in various industries, including the production of advanced optical coatings, due to their low weight, flexibility, and ease of processing [1,2]. However, their inherent limitations in thermal stability and optical performance restrict their use in harsh, high-temperature environments [3–5]. A promising strategy to overcome these challenges is the development of polymer nanocomposites, which offer multiple advantages [6,7]. This approach involves incorporating inorganic nanoparticles into a polymer matrix, resulting in materials with enhanced mechanical, thermal, and optical properties [8–11].

In recent decades, polymer nanocomposites have attracted significant interest because of their improved thermal and optical characteristics. For example, adding nanoparticles such as silica [12,13], titanium oxide [14,15], or carbon nanotubes [16,17] to various polymer matrices has shown enhancements in thermal stability and refractive index. Maleimide-based polymers are particularly noteworthy, as they inherently exhibit high glass transition temperatures (Tg) and excellent thermal stability, making them ideal candidates for high-performance applications [18–20].

Polymer nanocomposites exhibit remarkable optical, structural, and thermal properties that make them highly attractive for advanced applications. Optically, nanofillers can modulate the refractive index, reduce band gaps, and enhance light absorption or transparency, thereby enabling superior performance in coatings and optoelectronic devices [21–28,30]. Structurally, the incorporation of nanoparticles often reduces polymer crystallinity while improving dispersion and interfacial interactions, resulting in more amorphous phases that are suitable for flexible electronics and ion mobility [21,23,25,27,29]. Thermally, these materials demonstrate increased stability, higher decomposition temperatures, and greater heat resistance due to strong filler-matrix bonding [22,23,26,28]. Recent developments further highlight their growing importance in multifunctional systems, such as energy storage, sensors, protective coatings, and next-generation optoelectronics [28–31]. These synergistic improvements position nanocomposites as promising candidates for sustainable, high-performance materials across diverse industries [28,30,31].

In this context, layered materials have emerged as particularly promising nanofillers for nanocomposites [32], with zirconium phosphate (ZrP) being a notable example [33]. When properly dispersed, ZrP can enhance the properties of polymers due to its high thermal stability and extensive surface modifiability [34,35]. Previous studies aimed at optimizing the compatibility and interfacial adhesion of ZrP with polymers have utilized functionalization with organic groups, such as amines [36] and alkyl chains [37]. However, achieving an appropriate balance between high optical transmittance and thermal stability remains a significant challenge in high-performance optical coatings. While increased nanoparticle loading can enhance heat transfer properties, it often leads to aggregation and light scattering, which compromises optical transparency [38–41].

Despite advancements in ZrP-based nanocomposites achieved through amine [36] or alkyl [37] functionalization, these methods have notable limitations. Their reliance on relatively weak non-covalent interactions, such as hydrogen bonding or van der Waals forces, often leads to inadequate long-term dispersion stability, particularly at higher filler loadings. This commonly results in nanofiller aggregation, increased light scattering, and a significant reduction in optical transparency, thereby hindering the desired balance between thermal enhancement and optical clarity in advanced coatings [38–41]. To address these issues, the present study introduces thiol-functionalized ZrP (ZrP-SH), which facilitates strong covalent or hydrogen-bonding interactions with maleimide groups in the P(MMA-co-MI) matrix [42,43]. This approach significantly improves chemical compatibility and uniform nanoparticle dispersion, enabling simultaneous enhancements in optical transmittance and thermal stability while overcoming the shortcomings of previous functionalization strategies.

Building upon these foundations, this research reports the development of novel mechanically robust, transparent, and thermally stable nanocomposites based on poly(methyl methacrylate-co-N-2-methyl-4-nitrophenylmaleimide) (P(MMA-co-MI)) reinforced with thiol-modified zirconium phosphate (ZrP-SH). The primary innovation of this study lies in the thiol (-SH) functionalization of ZrP, which, unlike more conventional amine or alkyl modifications, provides strong and stable interfacial bonding with the P(MMA-co-MI) chains [42,43]. This enhancement improves chemical compatibility and nanoparticle dispersion, both of which are critical for achieving multifunctional polymer materials [44]. Ultimately, this modification is anticipated to resolve the challenge of simultaneously attaining high thermal stability and optical transparency, with the overarching goal of developing a new class of nanocomposites that outperform traditional silicate-based systems [45].

The selection of P(MMA-co-MI) as the polymer matrix is motivated by its combination of high optical transparency (derived from PMMA segments) and excellent inherent thermal stability (conferred by maleimide units with high Tg) [18–20]. Thiol-functionalized zirconium phosphate (ZrP-SH) was chosen as the nanofiller due to its layered α-ZrP structure, superior thermal resistance, large surface area for modification, and the ability of thiol groups to form strong covalent or hydrogen bonds with carbonyl-containing polymer chains [33–35,42,43]. This specific combination offers several key advantages, including enhanced interfacial compatibility, uniform dispersion at low loadings (≤1 wt%), substantial thermal improvements (e.g., a ~ 30 °C increase in Tg and an onset degradation temperature of ~297 °C), and preserved optical clarity—making it particularly well-suited for silicon-free advanced optical coatings that require both high transparency and thermal endurance.

The objectives of this research are as follows:

- To synthesize and incorporate ZrP-SH nanoparticles using a controlled approach that ensures high purity and optimal particle size for effective integration.
- To prepare P(MMA-co-MI)/ZrP-SH nanocomposites with varying ZrP-SH weight fractions and examine how nanofiller content influences material properties.
- To conduct comprehensive structural, thermal, and optical characterization, with a particular emphasis on achieving high transparency while enhancing thermal stability.

Ultimately, this study aims to advance the fundamental understanding of polymer-nanoparticle interactions and to develop novel materials suitable for next-generation optical coatings, high-precision sensors, and sophisticated optoelectronic devices.

## 2. Experimental details

### 2.1. Materials and methods

All chemicals were used as received unless otherwise stated. The following chemicals were sourced: zirconium(IV) chloride (ZrCl₄, ≥ 99.9% trace metals basis, Sigma-Aldrich), orthophosphoric acid (H₃PO₄, 85 wt%, Sigma-Aldrich), tetra-n-butylammonium hydroxide (TBA hydroxide, ~ 40 wt% in water, Sigma-Aldrich), nitric acid (70 wt%, Sigma-Aldrich), (3-mercaptopropyl)trimethoxysilane (≥95%, Sigma-Aldrich), methyl methacrylate (≥99%, Merck), benzoyl peroxide (BPO, ~ 75% with 25% water, Merck), 2-methyl-4-nitroaniline (≥99%, Merck), and maleic anhydride (≥99%, Merck). Ethyl acetate (≥99.5%, Merck) and methanol (≥99.5%, Merck) were distilled under vacuum over potassium hydroxide prior to use.

### 2.2. Synthesis of N-2-methyl-4-nitrophenylmaleimide (MI) monomer

The synthesis of the MI monomer was conducted in two sequential steps. In the first step, maleamic acid was prepared by dissolving 2-methyl-4-nitroaniline in 15 mL of acetone, followed by the addition of 7 g of maleic anhydride. The mixture was stirred at room temperature for 6 hours. Distilled water was then added, and stirring continued for an additional hour, resulting in the precipitation of yellowish maleamic acid. The product was isolated by filtration, dried at room temperature, and obtained in an 87% yield (15.5 g).

In the cyclization step, 10 g of the maleamic acid was dissolved in 15 mL of acetic anhydride, and 0.3 g of sodium acetate was added as a catalyst. The mixture was refluxed at 90 °C for 6 hours. Subsequently, the reaction mixture was added dropwise to an ice-water mixture over 4 hours while continuously stirring. The resulting cream-colored MI monomer precipitate was collected by filtration, dried, and isolated in a 98% yield (9.1 g) [46].

### 2.3. Synthesis of poly(MMA-co-MI) copolymer

The P(MMA-co-MI) copolymer was synthesized using free-radical polymerization. In a 250-mL round-bottom flask, 11 mL (0.1 mol) of methyl methacrylate (MMA) and 10 g (approximately 43 mmol) of the MI monomer were dissolved in 30 mL of ethyl acetate. Benzoyl peroxide (0.2 g) was added as the initiator, and the reaction mixture was heated to 80 °C for 8 hours under a nitrogen atmosphere. Upon completion of the reaction, the resulting polymer solution was precipitated in a large excess of cold methanol. The precipitate was collected by filtration, washed with methanol, and dried [46].

### 2.4. Synthesis and functionalization of ZrP-SH nanoparticles

The synthesis of zirconium phosphate (ZrP) began with the preparation of a zirconium solution. Zirconium tetrachloride (4.66 g, 20 mmol) was dissolved in 25 mL of water, yielding 5.51 g of zirconium oxychloride crystals (85.3% yield) and forming a saturated 2 M zirconium solution. Subsequently, 5 g of these crystals was reacted with 50 mL of 6 M orthophosphoric acid at 80 °C for 24 hours. The resulting ZrP precipitate was washed with demineralized water (DMW) and dried. Ion exchange was then performed by treating the precipitate with 20 mL of 0.1 M nitric acid for 1 hour, resulting in 7.12 g of ZrP (71.4% yield).

For the exfoliation step, 2.2 g of ZrP was added to 10 mL of 0.5 M aqueous tetra-n-butylammonium hydroxide (TBA) solution, producing a 0.5 M TBA-ZrP suspension. The mixture was stirred in an ice bath for 6 hours, followed by stirring at 25 °C for an additional 18 hours to achieve exfoliation. The exfoliated product was then isolated by filtration and dried.

**Fig 1:**
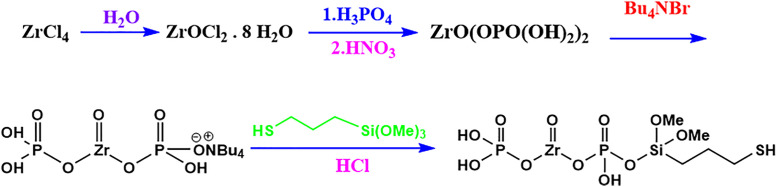
Schematic illustration of the two-step synthesis of thiol-functionalized zirconium phosphate (ZrP-SH) nanoparticles. The process involves the initial preparation of layered zirconium phosphate from zirconium tetrachloride, followed by surface functionalization with a thiol-containing silane coupling agent.

Thiol functionalization was conducted by treating the exfoliated ZrP with 0.4 mL of (3-mercaptopropyl)trimethoxysilane (SH-linker) dissolved in 4 mL of 0.1 M HCl. Specifically, 1 g of the TBA-exfoliated ZrP suspended in 5 mL of 0.1 M HCl was added dropwise to the SH-linker solution. The mixture was stirred at 65 °C for 1 hour, and the final ZrP-SH nanoparticles were collected by filtration. The complete synthesis and functionalization process of ZrP-SH nanoparticles is schematically illustrated in Fig 1.

### 2.5. Preparation of p(MMA-co-MI)/ZrP-SH nanocomposites.

The nanocomposites containing 0.3, 0.5, 0.7, and 1 wt% ZrP-SH were prepared using a solution precipitation method. For each composition, the appropriate amount of ZrP-SH was dispersed in 5 mL of ethyl acetate via sonication for 20 minutes. Separately, a polymer solution was created by dissolving 1 g of P(MMA-co-MI) in ethyl acetate. After the ZrP-SH was fully dispersed, the nanoparticle dispersion was added to the polymer solution, and the resulting mixture was sonicated for an additional 20 minutes to ensure thorough mixing. The combined solution was then precipitated in a large excess of methanol. The precipitated nanocomposite was collected by filtration, washed with methanol, and dried.

## 3. Characterization

The synthesized materials were characterized using a range of advanced analytical techniques.

Fourier-transform infrared (FTIR) spectra were recorded on a Bruker spectrometer using KBr disks over the wavenumber range of 4000–400 cm ⁻ ¹.

The morphology and nanofiller dispersion were investigated through scanning electron microscopy (SEM) operated at 5 kV and transmission electron microscopy (TEM) using a Philips 208 S instrument at 75 kV.

The crystalline structure was analyzed by X-ray diffraction (XRD) on a Bruker D8 ADVANCE diffractometer with Cu Kα radiation (λ = 1.5406 Å). Data were collected in the 2θ range of 5–50° at a scan rate of 2°/min with a step size of 0.02°.

Thermal stability was assessed using thermogravimetric analysis (TGA) with a TA Instruments Q2000 under a nitrogen atmosphere at a heating rate of 20 °C/min.

Glass transition temperatures (Tg) were determined by differential scanning calorimetry (DSC) with a TA Q2000 under a nitrogen atmosphere, using a heating rate of 20 °C/min from 80 to 150 °C and a cooling rate of 10 °C/min.

Optical transparency was quantitatively evaluated using a Photonix Ar 2015 UV-Vis spectrophotometer. Measurements were performed on solutions prepared by dissolving 50 mg of the nanocomposite in 10 mL of ethyl acetate, with agitation on a rotary shaker.

## 4. Result

The present study successfully synthesized a novel series of P(MMA-co-MI)/ZrP-SH nanocomposites reinforced with thiol-functionalized zirconium phosphate (ZrP-SH) nanofillers. The results show that incorporating these nanofillers at optimal loadings significantly enhances the material properties, as demonstrated by comprehensive characterization. The findings not only establish a baseline for the performance of the functionalized nanofiller but also achieve the stated objectives by clarifying the interfacial interactions within the polymer matrix.

### 4.1. Characterization of synthesized nanoparticles

**Fig 2:**
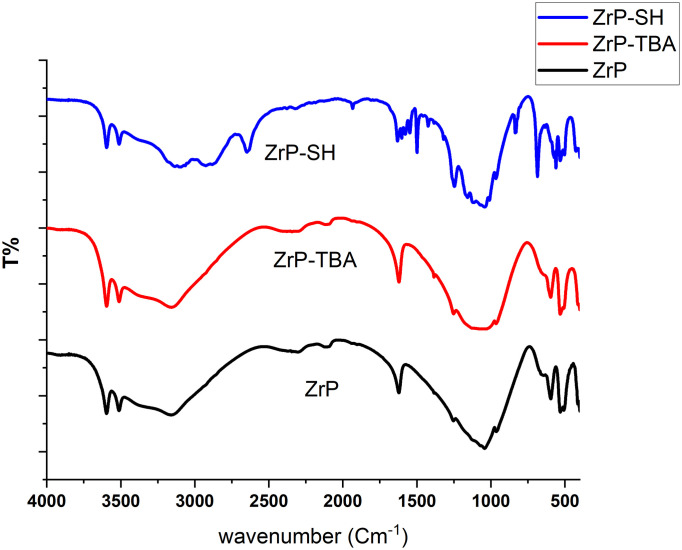
FTIR spectra of pristine ZrP, ammonium salt-modified ZrP (ZrP-TBA), and thiol-functionalized ZrP (ZrP-SH) nanoparticles. The spectra confirm the successful surface modification of the nanoparticles through the various functionalization steps.

Fourier-transform infrared (FTIR) spectroscopy confirmed the successful thiol functionalization of ZrP (Fig 2). The spectrum of pristine ZrP exhibited characteristic P–O stretching at approximately 1050 cm ⁻ ¹ and broad O–H bands around 3500 cm ⁻ ¹. Following TBA exfoliation (ZrP-TBA), alkyl C–H stretching bands appeared in the range of 2850–2950 cm ⁻ ¹. In the case of ZrP-SH, a weak S–H stretching band emerged at approximately 2550 cm ⁻ ¹, accompanied by a reduction in O–H band intensity, thereby verifying thiol attachment. These spectral changes provide direct evidence of effective surface modification, which is essential for subsequent interfacial interactions in the nanocomposites [46].

**Fig 3:**
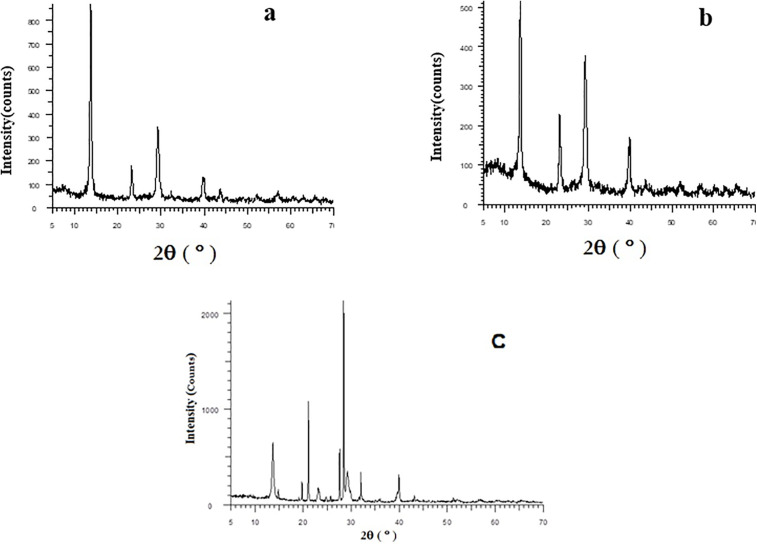
Xray diffraction (XRD) patterns of zirconium phosphate nanoparticles. **(a)** Pristine ZrP, (b) ammonium salt-modified ZrP (ZrP-TBA), and (c) thiol-functionalized ZrP (ZrP-SH). The appearance of new peaks and changes in peak intensities confirm successful intercalation and surface functionalization.

XRD analysis further validated these structural modifications (Fig 3). The diffraction pattern of pristine ZrP displayed characteristic peaks for its layered structure at approximately 2θ = 7°, 14°, and 23°, consistent with its known crystalline structure. The ZrP-TBA pattern retained a similar overall profile but exhibited significant broadening and decreased intensity of the peak at 2θ = 14°. These changes indicate the intercalation of the bulky ammonium salt, disruption of long-range crystalline order, and increased interlayer spacing.

The ZrP-SH pattern revealed a distinct profile, with new peaks emerging at approximately 2θ = 20°, 21°, 27.5°, 28.5°, and 32°, alongside residual peaks from the original ZrP. These observations provide strong evidence for the successful formation of the hybrid ZrP-SH nanoparticles and confirm structural alterations resulting from covalent thiol attachment. The average crystallite size, calculated using the Scherrer equation (D = Kλ / (β cos θ)), decreased from 191.3 Å for pristine ZrP to 103.6 Å for ZrP-TBA and subsequently recovered to 190.8 Å for ZrP-SH. This recovery demonstrates effective preservation and control of the crystalline structure during the thiolation process.

### 4.2. Morphological and thermal characterization of ZrP-SH nanoparticles.

**Fig 4:**
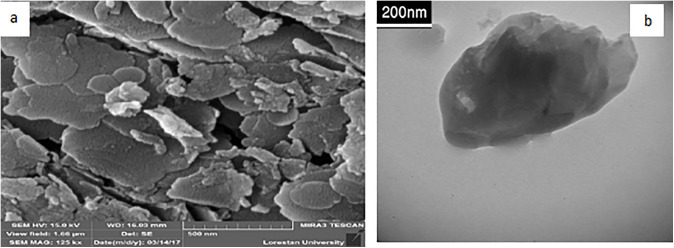
Morphological characterization of thiol-functionalized ZrP-SH nanoparticles. **(a)** Scanning electron microscopy (SEM) image revealing plate-like morphology and (b) transmission electron microscopy (TEM) image confirming nanoscale dimensions.

To confirm that the layered structure remained intact during synthesis, we thoroughly examined the morphology of the ZrP-SH nanoparticles. The ZrP-SH nanoparticles exhibited a characteristic plate-like or flaky morphology, consistent with their layered crystalline structure, as illustrated in the SEM and TEM images (Fig 4). The SEM image (Fig 4a) provided a top-down view of the particles, revealing a uniform shape and size distribution with a high degree of ordering. The TEM image (Fig 4b) offered a detailed view of individual nanoparticles, further confirming their uniformity, nanoscale dimensions, and preserved crystallinity. This high-surface-area morphology is expected to enhance interfacial interactions in the final polymer matrix, which is critical for optimizing the performance of polymer nanocomposites.

### 4.3. Thermal characterization of synthesized nanoparticles.

**Fig 5:**
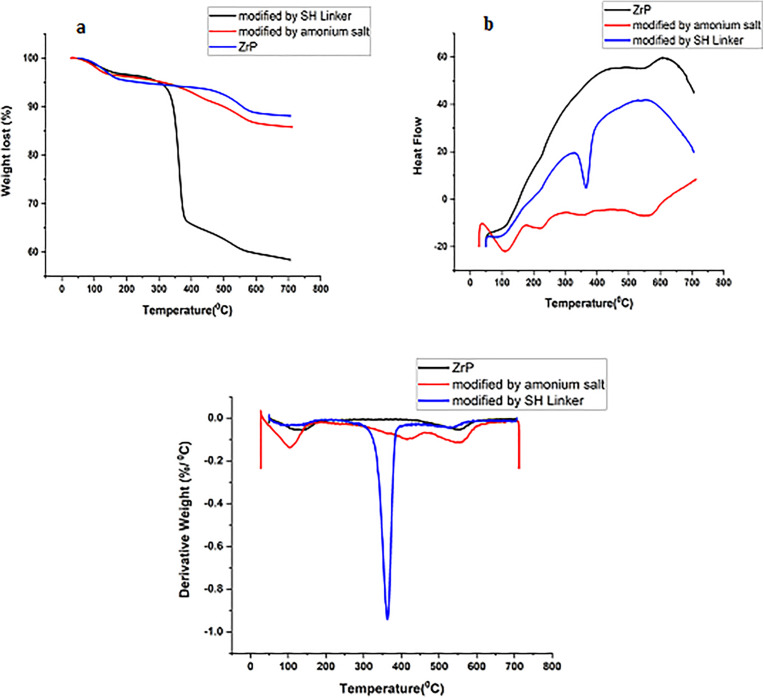
Thermal analysis of pristine ZrP, ammonium salt-modified ZrP (ZrP-TBA), and thiol-functionalized ZrP (ZrP-SH). **(a)** Thermogravimetric analysis (TGA) curves, (b) differential scanning calorimetry (DSC) curves, and (c) derivative thermogravimetric (DTG) curves, demonstrating successful attachment and thermal decomposition of the thiol linker.

Thermal analysis (TGA, DSC, and DTG) provided further evidence of successful surface modification (Fig 5). The TGA curve for pristine ZrP (Fig 5a) showed an initial weight loss due to moisture below 100 °C, followed by a gradual loss attributed to the dehydration of interlayer water at higher temperatures. In contrast, ZrP-SH exhibited a sharp and pronounced weight loss between 300 and 400 °C, which is markedly different from the pristine ZrP curve. This weight loss is ascribed to the thermal decomposition of the covalently attached thiol linker, explaining the observed differences.

Complementary DTG (Fig 5c) and DSC (Fig 5b) analyses revealed sharp exothermic peaks in the same temperature range, further supporting the successful covalent attachment and the thermal stability of the modifying groups. These results provide direct thermal evidence of functionalization, thereby validating the synthetic strategy.

### 4.4. Characterization of p(MMA-co-MI)/ZrP-SH nanocomposites.

#### 4.4.1. Structural characterization.

**Fig 6:**
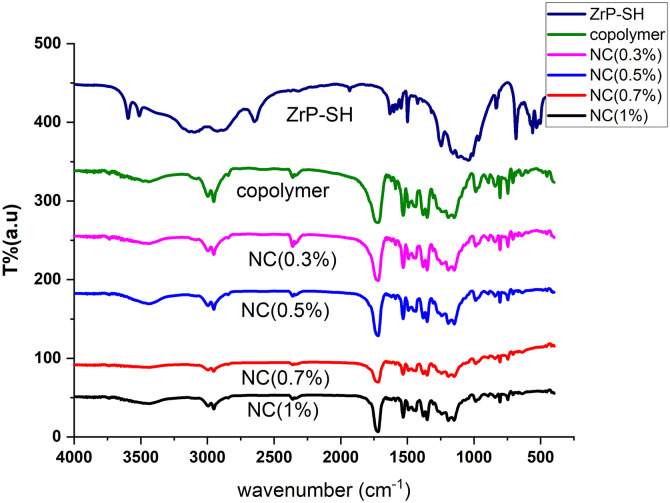
FTIR spectra of ZrP-SH nanoparticles, pristine P(MMA-co-MI) copolymer, and P(MMA-co-MI)/ZrP-SH nanocomposites. The spectra for nanocomposites containing 0.3, 0.5, 0.7, and 1 wt% nanofiller confirm the incorporation of both components in the hybrid materials.

Fourier-transform infrared (FTIR) spectra of the P(MMA-co-MI)/ZrP-SH nanocomposites (Fig 6) confirmed the successful formation of hybrid materials, as indicated by the coexistence of characteristic peaks from both the copolymer and the ZrP-SH nanofiller. The spectra displayed P–O stretching from ZrP-SH at approximately 1050 cm ⁻ ¹, along with key copolymer bands, including C = O (ester/maleimide) stretching at around 1725 cm ⁻ ¹ and C–N stretching at approximately 1380 cm ⁻ ¹. Notably, the C = O peak exhibited a red shift to lower wavenumbers with increasing ZrP-SH loading, suggesting hydrogen bonding or possible covalent interactions between the thiol groups on the nanoparticle surface and the carbonyl oxygen atoms in the maleimide units of the copolymer. The intensity of the P–O peak increased proportionally with filler content without significant broadening, indicating a uniform distribution of the nanofiller and minimal aggregation. These spectral features provide strong evidence of robust interfacial adhesion facilitated by the thiol functionalization strategy, which is essential for the observed improvements in thermal stability and optical properties.

#### 4.4.2. Morphological characterization and nanofiller dispersion.

**Fig 7:**
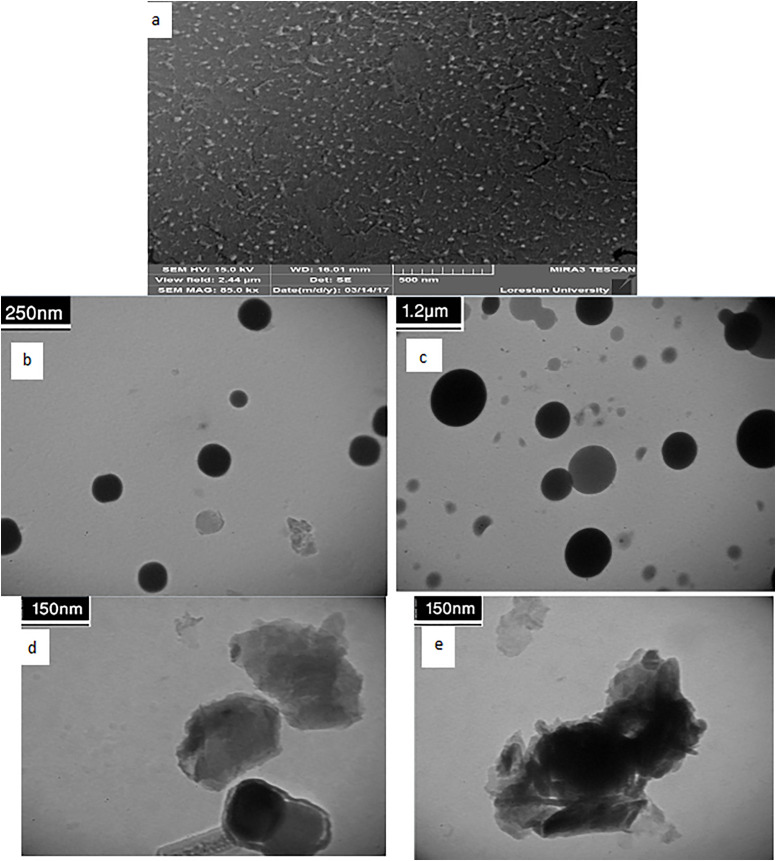
Morphological and dispersion analysis of thiol-functionalized ZrP-SH nanoparticles in P(MMA-co-MI) nanocomposites. **(a)** SEM image of the 0.3 wt% nanocomposite showing a smooth fractured surface with homogeneous dispersion; **(b, c)** TEM images of the 0.5 wt% and 0.3 wt% nanocomposites revealing well-dispersed individual nanoparticles; **(d, e)** TEM images of the 0.7 wt% and 1 wt% nanocomposites indicating agglomeration at higher loadings; and (f) selected-area electron diffraction (SAED) pattern confirming the crystalline structure of ZrP-SH nanoparticles.

The dispersion of the nanofiller within the polymer matrix, along with its morphological integrity, was investigated using scanning electron microscopy (SEM) and transmission electron microscopy (TEM). The SEM image of the 1 wt% nanocomposite (Fig 7a) revealed a smooth and homogeneous fractured surface, with no evidence of large-scale aggregates or clusters—an important indicator of effective dispersion.

TEM images of the lower-loading nanocomposites (0.3 and 0.5 wt%) further supported these findings (Fig 7b,c). The micrographs showed individual ZrP-SH nanoparticles uniformly distributed throughout the polymer matrix without agglomeration. This homogeneous dispersion can be attributed to the optimized thiol functionalization, which enhanced compatibility between the hybrid nanofiller and the organic polymer chains. Additionally, selected-area electron diffraction (SAED) patterns of the nanoparticles within the composites exhibited sharp, bright spots (Fig 7f), confirming the preservation of their crystalline structure and integrity after incorporation into the polymer matrix.

As expected, higher nanofiller loadings (0.7 and 1 wt%) led to the onset of agglomeration and irregular aggregate formation, as observed in the corresponding TEM images (Fig 7d,e). These features underscore the importance of controlling nanofiller concentration during processing to achieve the desired balance of properties.

#### 4.4.3. Enhanced thermal properties.

**Fig 8:**
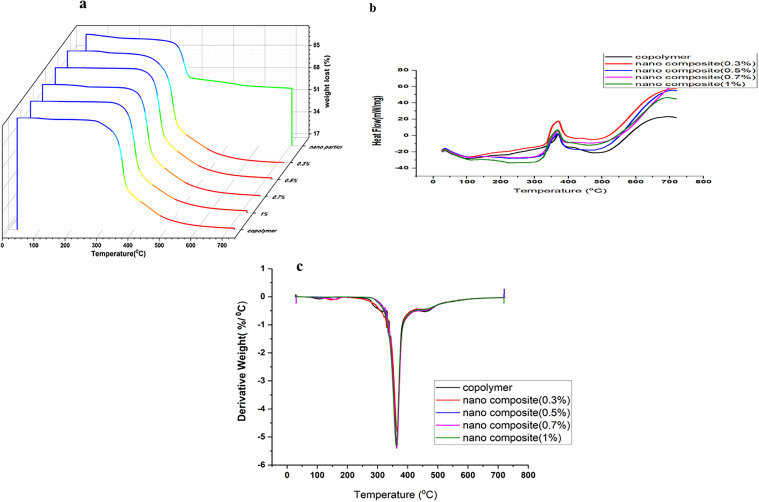
Thermal analysis of the pristine P(MMA-co-MI) copolymer and its ZrP-SH nanocomposites. **(a)** Thermogravimetric analysis (TGA) curves, (b) differential scanning calorimetry (DSC) curves, and (c) derivative thermogravimetric (DTG) curves for various nanofiller loadings.

Our thermal analyses, summarized in Table 1 and illustrated by the TGA and DSC curves (Fig 8), reveal a significant and unprecedented improvement in the thermal stability of the material. This finding addresses one of the major challenges in developing high-performance polymer nanocomposites.

**Table 1:** A summary of the thermal properties (including TGA and DSC data) for the pristine P(MMA-co-MI) copolymer and its ZrP-SH nanocomposites at various nanofiller concentrations.

Sample designation	Copolymer	NC-0.3	NC-0.5	NC-0.7	NC-1
Nanoparticle%	0	0.3	0.5	0.7	1
T_10_	327.5	322.3	343.4	341.8	341.1
T_50_	369.9	372.6	374.7	374.5	373.7
T_max_	576.18	682.6	667.2	663.6	678
T_g_	133.7	164.3	164.5	164.5	168.4
Char Yield	15.3	14.6	15.99	15.5	16.4

The TGA results (Fig 8a) indicate that the thermal degradation onset temperature of the pristine P(MMA-co-MI) copolymer increased from 265 °C to approximately 297 °C upon incorporation of the ZrP-SH nanofiller. Additionally, the temperature corresponding to 50% weight loss (T₅₀), which was 369.9 °C for the pristine copolymer, shifted to a range of 372.6–374.7 °C in the nanocomposites. Remarkably, the final degradation temperature for the nanocomposites reached nearly 670 °C, compared to only 576.2 °C for the pristine copolymer.

The DSC data (Fig 8b) further confirm strong polymer–nanofiller interactions. The glass transition temperature (T_g) of the pristine copolymer was 133.7 °C but increased significantly to between 164.3 and 168.4 °C upon the addition of ZrP-SH. This increase of more than 30 °C reflects a considerable restriction of polymer chain mobility, attributable to intense chemical and physical interactions at the interface, facilitated by the thiol functionalization of the ZrP nanoparticles. These results underscore the effectiveness of the synthetic strategy and the relative ease with which a high-performance hybrid material can be produced.

Further insight into the thermal degradation process is provided by the DTG curves (Fig 8c). The pristine copolymer exhibited its main degradation peak at approximately 350 °C, whereas the nanocomposites displayed this peak in a higher temperature range of 355–365 °C. This shift provides additional evidence of enhanced thermal stability resulting from the well-dispersed ZrP-SH nanofiller.

#### 4.4.4. Tunable optical performance.

**Fig 9:**
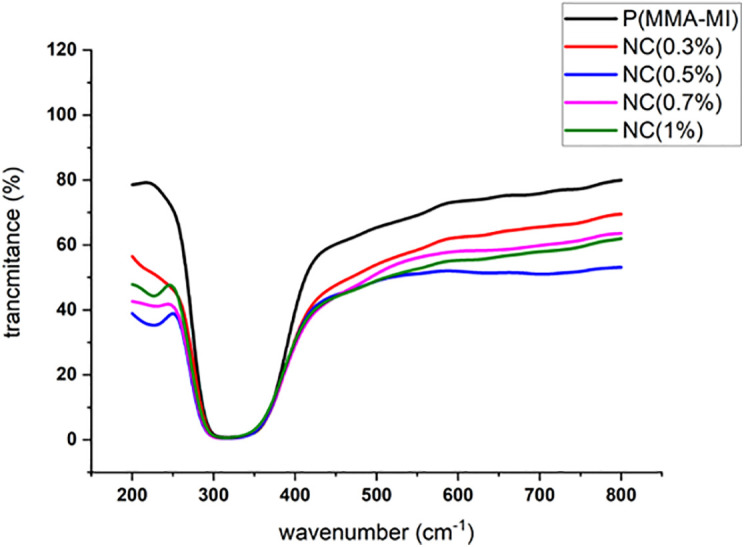
UV-Vis transmittance spectra of the pristine P(MMA-co-MI) copolymer and its ZrP-SH nanocomposites. The spectra at varying nanofiller loadings illustrate maintained high transparency in the visible region despite increasing filler content.

The UV-Vis spectroscopy results, presented in Fig 9, demonstrate that the nanocomposites retained excellent optical transparency, successfully overcoming the common trade-off between enhanced thermal stability and optical clarity in polymer nanocomposites. The pristine P(MMA-co-MI) copolymer exhibited a high transmittance of approximately 80% in the visible range (400–700 nm). Upon incorporation of ZrP-SH nanoparticles, transmittance gradually decreased with increasing nanofiller concentration, particularly beyond 0.3 wt%. This reduction is expected, as nanoparticles can scatter visible light and thus reduce light transmission.

Nevertheless, the nanocomposites maintained remarkably high transparency, especially at lower nanofiller loadings. This superior optical clarity results from the uniform and homogeneous dispersion of ZrP-SH nanoparticles within the polymer matrix, as evidenced by our SEM and TEM analyses. The absence of large aggregates minimizes light scattering, thereby preserving optical performance. Consequently, precise control over nanofiller concentration allows for tuning of the optical properties to meet specific application requirements—a critical feature for developing high-performance materials in targeted optical applications.

As highlighted in preceding sections, the successful synthesis of P(MMA-co-MI)/ZrP-SH nanocomposites has yielded materials that combine exceptional thermal stability with tunable optical performance, positioning them at the forefront of innovation in materials science and engineering.

## 5. Discussion

The results of this study elucidate the intricate interplay between nanofiller functionalization and dispersion in determining the properties of polymer nanocomposites. FTIR analysis (Fig 2) and TGA data (Fig 5) confirm the successful thiol functionalization of zirconium phosphate (ZrP-SH), enhancing compatibilization between the organic P(MMA-co-MI) matrix and the inorganic nanofiller—a critical factor for achieving optimal performance in polymer nanocomposites. This improved compatibilization is particularly evident at low filler concentrations, as demonstrated by the uniform and homogeneous dispersion of ZrP-SH nanoparticles in the SEM and TEM images (Fig 4). The absence of significant agglomeration can be directly attributed to the functionalization strategy, which is essential for realizing synergistic property enhancements.

The integrated application of SEM/TEM, XRD, and TGA provides comprehensive insights into the nanocomposite structure and performance. SEM and TEM offer direct visual evidence of nanofiller morphology and dispersion, revealing a uniform platelet-like distribution of ZrP-SH with minimal aggregation at low loadings (Figs 4 and 7). This behavior arises from enhanced interfacial adhesion facilitated by thiol functionalization. XRD analysis complements these observations by confirming structural changes, such as altered interlayer spacing and reduced polymer crystallinity resulting from intercalation/exfoliation (Fig 3), which further supports the homogeneous dispersion seen microscopically. TGA measurements quantify the resultant thermal improvements, including elevated degradation onset temperatures and restricted chain mobility (Figs 5 and 8). Collectively, these techniques establish a clear correlation between nanoscale interfacial interactions (thiol–maleimide bonding) and macroscopic property enhancements, thereby validating the efficacy of the functionalization approach.

**Fig 10:**
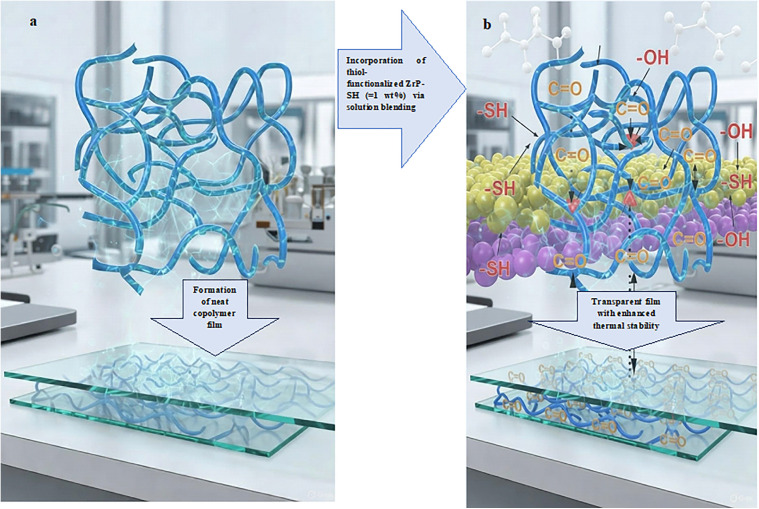
Schematic illustration of the reinforcement mechanism and interfacial interactions in P(MMA-co-MI)/ZrP-SH nanocomposites. **(a)** Neat P(MMA-co-MI) copolymer chains forming a transparent film; (b) incorporation of thiol-functionalized ZrP-SH nanoparticles into the copolymer matrix. Strong hydrogen bonding between the –SH/–OH groups on ZrP-SH and carbonyl groups in the maleimide units ensures uniform nanofiller dispersion at low loadings ($\le$1 wt%), preserving high visible-light transparency and enhancing thermal stability. These silicon-free nanocomposites show promise for applications in advanced optical coatings, sensors, protective layers, and optoelectronic packaging.

Furthermore, the retention of high optical transmittance (Fig 9) demonstrates that strong interfacial adhesion does not compromise optical clarity, successfully addressing a common trade-off in nanocomposite materials. The observed enhancements in thermal stability, coupled with preserved optical transparency, are primarily attributable to robust interfacial interactions between the thiol-functionalized ZrP-SH nanoparticles and the P(MMA-co-MI) matrix, as schematically depicted in Fig 10. The thiol (–SH) and residual hydroxyl (–OH) groups on the ZrP-SH surface form strong hydrogen bonds with the carbonyl (C = O) groups in the maleimide units, thereby restricting polymer chain mobility and promoting homogeneous dispersion even at low filler loadings (≤1 wt%). These results underscore the effectiveness of the thiol functionalization strategy in achieving well-balanced property improvements.

### 5.1. Mechanistic understanding of property enhancement.

Substantial evidence supports the formation of strong interfacial interactions between the functionalized ZrP-SH nanoparticles and the P(MMA-co-MI) polymer chains. These interactions primarily involve hydrogen bonding between the carbonyl groups of the maleimide units in the copolymer and the hydroxyl (–OH) groups on the ZrP-SH surface, as indicated by the observed red-shift in the FTIR spectra. This bonding restricts polymer segmental motion, resulting in a more rigid structure that enhances thermal resistance. This mechanism primarily explains the significant increase in glass transition temperature (Tg).

From a practical perspective, these improvements demonstrate the suitability of the synthesized nanocomposites for demanding applications, including high-performance optical coatings, electronic encapsulation, and structural components in thermally challenging environments.

The UV-Vis spectroscopy results reveal a gradual decrease in optical transmittance with increasing nanofiller content (Fig 9), allowing for precise tuning of the optical properties. This tunability is particularly valuable for designing materials intended for specific optical applications that require controlled transmission or absorption of light at targeted wavelengths. The observed reduction in transparency at higher filler loadings aligns with established theories of light scattering in composite materials, where the refractive index mismatch between the nanofiller and the polymer matrix leads to increased scattering and reduced light transmission.

### 5.2. Advancing the field: novelty and contribution

This work closely aligns with established findings in the literature while providing novel and detailed insights into design principles for polymer nanocomposites. It is widely accepted that well-dispersed inorganic nanoparticles enhance the thermal stability and glass transition temperature (Tg) of polymer matrices; however, the present study offers specific empirical evidence for a previously unreported material system.

In particular, we demonstrate—for the first time—the effectiveness of thiol-functionalized zirconium phosphate (ZrP-SH) in a P(MMA-co-MI) matrix. Achieving uniform dispersion at low filler loadings through thiol functionalization exemplifies an efficient surface modification strategy. Consequently, this research refines the fundamental understanding of how specific functional groups promote strong interfacial interactions between layered inorganic fillers and complex copolymers, resulting in predictable and substantial property enhancements that contribute new insights to the field.

### 5.3. Limitations and future directions

While this study has achieved significant progress, several limitations should be acknowledged, along with corresponding opportunities for future research. The investigation primarily focused on the structural, thermal, and optical characterization of the nanocomposites, and did not include a comprehensive assessment of their mechanical properties, such as tensile strength, Young’s modulus, and toughness. Conducting direct experimental mechanical testing in future work would yield valuable insights into practical applications.

Additionally, optical transparency was evaluated only in solution, which may not accurately represent performance in more application-relevant solid films or coatings. Therefore, future studies should prioritize direct measurement of optical properties in solid films.

Although device-level experimental validation (e.g., fabrication of thin-film optical coatings and thermal cycling tests) was beyond the scope of this fundamental materials development study, the substantial property improvements observed provide strong foundational support for the proposed applications. Such applied testing is planned for subsequent investigations.

Furthermore, exploring a wider range of filler concentrations with finer increments would enable more precise optimization of loading levels for targeted property enhancements. Lastly, long-term stability and resistance to environmental degradation (e.g., under UV radiation, humidity, or chemical exposure) were not examined in this work. Incorporating accelerated aging tests in future studies would help confirm durability and ensure reliable performance in real-world applications.

### 5.4. Practical relevance, and potential applications

Although previous studies have reported property enhancements in ZrP-based polymer nanocomposites—such as improved thermal stability or dispersion achieved through amine- or alkyl-functionalization in epoxy or other matrices—this work introduces a distinct advance by employing thiol-functionalized zirconium phosphate (ZrP-SH) in a transparent maleimide-containing copolymer. To our knowledge, this is the first demonstration of ZrP-SH providing superior interfacial compatibility and uniform dispersion at very low loadings (≤1 wt%), resulting in substantial improvements in thermal stability (∼30 °C increase in Tg and onset of degradation at ∼297 °C) while preserving high optical transparency.

These balanced property enhancements not only represent a fundamental advance but also open up promising practical applications, particularly in advanced optical coatings and silicon-free transparent barriers. The combination of high visible-light transmittance (>90% at optimal low loadings, Fig 9) and significantly elevated thermal endurance directly addresses critical performance gaps in conventional polymers, which often suffer from degradation or light scattering under thermal stress.

The nanocomposites meet or exceed established requirements for several targeted applications. For protective optical coatings in optoelectronic devices (e.g., LED encapsulants or high-power optics), industry benchmarks typically demand visible transmittance >85–90% and thermal stability sufficient for operation up to 200–250 °C without significant degradation. The present materials, at ≤1 wt% ZrP-SH, achieve transmittance >90% (Fig 9), Tg > 160 °C, and onset degradation at ∼297 °C (Figs 5 and 8), while the silicon-free composition and homogeneous dispersion (Figs 4 and 7) minimize haze and enhance long-term reliability compared to silicone-based alternatives.

In optical sensors for harsh environments, controllable transparency and resistance to thermal cycling are essential. The UV-Vis spectra show tunable transmittance with filler loading (Fig 9), complemented by the ∼30 °C Tg increase and improved thermal stability (DSC/TGA data), ensuring operational reliability under elevated temperatures and repeated thermal stress.

For advanced packaging requiring transparent barrier layers, high optical clarity, low haze, and superior thermal durability are paramount. The uniform nanoparticle dispersion at low loadings effectively suppresses light scattering while delivering significant thermal enhancements, outperforming conventional PMMA- or silicate-based systems.

Overall, these results establish the P(MMA-co-MI)/ZrP-SH nanocomposites as viable candidates that effectively resolve the common thermal-optical trade-off. The findings distinguish this system from earlier ZrP composites primarily optimized for mechanical reinforcement or catalysis. Future investigations involving prototype thin-film fabrication, thermal cycling tests, and device-level integration are recommended to confirm real-world performance.

## 6. Conclusion

This study reports the development of transparent and thermally stable nanocomposites based on poly(methyl methacrylate-co-N-(2-methyl-4-nitrophenyl)maleimide) (P(MMA-co-MI)), reinforced with thiol-functionalized zirconium phosphate (ZrP-SH). Thiol functionalization and uniform dispersion of the nanofiller at low loadings (≤1 wt%) were confirmed through spectroscopic, microscopic, and thermal analyses.

Key findings include the preservation of high visible-light transmittance (>90% at low filler contents), an increase in glass transition temperature of approximately 30 °C (rising to 164.3–168.4 °C), and an increase in onset degradation temperature to around 297 °C. These enhancements are attributed to strong interfacial interactions between the thiol groups on ZrP-SH and the maleimide units in the copolymer matrix.

The resulting nanocomposites effectively balance optical transparency and thermal stability at low filler loadings, offering significant advantages for silicon-free applications, including advanced optical coatings, sensors, protective layers, and optoelectronic packaging. The thiol-functionalization strategy presents a promising approach for designing multifunctional polymer nanocomposites.

Future work should explore mechanical properties, long-term environmental durability, and device-level performance to facilitate practical implementation.
